# Anesthesia Management in Emergency and Trauma Surgeries: A Narrative Review

**DOI:** 10.7759/cureus.66687

**Published:** 2024-08-12

**Authors:** Taysir M Alnsour, Mohammed A Altawili, Arwa M Alhoqail, Faisal Y Alzaid, Yousef O Aljeelani, Areej M Alanazi, Rakan K Alfouzan, Sultan Alsultan, Abdulrahman A Almulhem

**Affiliations:** 1 Anaesthesiology, King Fahad Specialist Hospital, Tabuk, SAU; 2 General Practice, Alaziziyah Primary Health Care Center, Tabuk, SAU; 3 General Practice, National Guard Health Affairs, Riyadh, SAU; 4 General Practice, King Saud Bin Abdulaziz University for Health Sciences, Riyadh, SAU; 5 Anesthesiology, Taibah University, Madinah, SAU; 6 Pediatrics, Northern Border University, Arar, SAU; 7 General Practice, King Salman Hospital, Riyadh, SAU; 8 Anesthesiology, National Guard Health Affairs, Riyadh, SAU; 9 Anesthesiology, King Faisal University, Al-Ahsa, SAU

**Keywords:** surgery, trauma surgery, emergency, sedation, anesthesia

## Abstract

Emergency and trauma surgeries present unique challenges for anesthesiologists due to the acuity of patient conditions and the need for rapid intervention. This review aims to provide insights into the optimal management of anesthesia in emergency and trauma surgery settings. We searched the National Institute of Health PubMed, Scopus, MEDLINE, and Web of Science databases between 2014 and 2024 to synthesize current evidence and best practices for anesthesia management during emergency and trauma surgeries. This literature review examines the evolving role of anesthesia in emergency and trauma surgeries, focusing on key considerations such as patient management, hemodynamic stability, and the choice of anesthetic agents. The review discusses recent advancements in anesthesia techniques, including the use of regional anesthesia and multimodal analgesia, to optimize patient outcomes while minimizing complications. Additionally, it discusses the importance of interdisciplinary collaboration among anesthesiologists, surgeons, and other healthcare professionals in delivering timely and effective care to critically injured patients.

## Introduction and background

Trauma is a serious public health concern. In the United States (US), trauma accounts for 30% of all life years lost; this is greater than the combined effects of HIV, heart disease, and cancer. It is considered the third overall and the primary cause of death for people between the ages of 1 and 44 years [1]. However, there is a notable lack of comprehensive, official global data on trauma. This gap in data is significant because understanding the worldwide impact of trauma is crucial for developing effective public health strategies. Therefore, we call for the collection and dissemination of global trauma statistics to better inform international health policies and interventions.

Although middle-aged patients experience a higher level of morbidity due to trauma than other age groups, the elderly have a significantly higher level of morbidity due to minor trauma and increased levels of frailty [2]. In the USA alone, the annual cost of trauma surpassed $400 billion in 2019 [3]. In 2020, the fourth leading cause of death was due to unintentional injuries [4]. Post-traumatic stress disorder, which affects veterans, active-duty military personnel, and civilians, is estimated to cost the US economy $232.2 billion annually [5]. Due to injuries from trauma continuing to be a large proportion of emergency department visits, prompt performance and decision-making are essential in a short time to preserve the patient's life.

Anesthesiology is a specialized branch of medicine practiced by doctors with advanced training. "The practice of medicine dedicated to the relief of pain and total care of surgical patients before, during, and after surgery" is how the American Society of Anesthesiologists defines anesthesiology [6]. An anesthesiologist is a highly trained specialist physician, in anesthesia alone or combined with intensive care, who treats patients continuously before, during, and following surgery so they can resume normal anatomical, physiological, pharmacological, and psychological functions [7]. They also provide emergency medical services extending from the injury scene through transportation to the surgery site within the institution [8]. They exert a large part of their efforts on complex patients. They also handle the patient's surgical needs and post-trauma rehabilitation [9]. Trauma anesthesiologists offer a unique set of skills in the multidisciplinary treatment of trauma patients because they can act quickly and effectively in a short time, which is crucial to improving patient outcomes.

Anesthesia for emergencies is not scheduled, so there is not much time to assess the patient preoperatively and prepare for the operation. Because of the patient's potential for rapid deterioration, prompt performance and decision-making are essential in a short time to preserve the patient's life [10].

We aimed to summarize the role of anesthesia in emergency and trauma surgeries to provide a comprehensive understanding of current practices and outcomes related to anesthesia administration in these critical settings.

## Review

Methodology

Searches were made on the National Institute of Health PubMed, Scopus, MEDLINE, and Web of Science databases between 2014 and 2024, using the phrases ("Anesthesia" OR "Anaesthesia" OR “sedation”) AND "management") AND ("Emergency" OR "Trauma Surgery"). We included 74 studies discussing the role of anesthesia in emergency and trauma surgery to provide a comprehensive understanding of current practices and outcomes related to anesthesia administration in these critical settings. This could involve examining various anesthesia drugs used, patient outcomes, complications, and strategies for optimizing anesthesia management to improve patient care and surgical outcomes in emergency and trauma situations. There were no limitations regarding study designs.

The role of anesthesiologists in emergency medicine

Emergency surgery refers to procedures that call for an immediate hospital admission, typically via the accident and emergency departments. Emergency surgery can be done immediately or throughout the night for conditions that are serious or life-threatening, and it is often completed within 24 hours [11]. Unlike elective anesthesia, emergency anesthesia requires higher service demands. The severity of the illness, the need for quick management, and the number of cases cause the demands to vary unexpectedly. The unpredictability of emergency anesthesia, coupled with the frequent lack of information about patients' comorbidities and chronic medications, makes it more difficult to deliver care that satisfies accepted standards of practice. Therefore, hospitals must have enough space and adaptable mechanisms to handle changes in patient demand and illness severity [12].

When managing emergencies, surgeons, anesthesiologists, and other medical specialists must coordinate their logistical efforts closely. Emergency surgeries take precedence over all other cases, and anesthesia may be administered in desperate circumstances such as severe abdominal, cardiac, thoracic, or major vascular injuries and obstetric or pediatric emergencies [13]. Anesthesia for emergencies is administered in many settings. Depending on the setting and organization, emergency physicians or paramedics are responsible for airway instrumentation and sedation/anesthesia in prehospital emergency departments (EDs) [14].

The role of anesthetic drugs in pain control in the ED

Every anesthesiologist has to deal with unexpected, life-threatening emergencies. Anesthesiologists play a crucial role in managing pain during emergencies. For immediate surgical intervention in emergency cases, the use of proper anesthesia is necessary. It helps to prevent the painful status of surgery and helps the surgical team avoid dealing with an agitated and uncooperative patient, thus decreasing the risk of multiple complications. Anesthesia serves as a preventative measure in these high-risk situations [15]. Numerous non-opioid substitutes have been widely used in the ED with excellent results in treating a wide range of painful conditions, as evidenced by a substantial body of literature. According to the American College of Emergency Physicians and the American Academy of Emergency Medicine, ketamine is a powerful analgesic and non-competitive antagonist of the N-methyl-D-aspartate (NMDA)/glutamate receptor complex that can be used in the ED to treat both acute and chronic pain [16]. When administered in sub-dissociative (SDK) doses, the common IV dosing regimen is 0.1 to 0.3 mg/kg, or a 15 to 30 mg fixed dose given over 15 minutes to reduce psycho-perceptual adverse effects [17-19]. It has been demonstrated that to treat pain in the ED, SDK at 0.3 mg/kg IV is equally effective as morphine at 0.1 mg/kg IV [18-20]. Without IV access, SDK can be given intranasal (IN) at 0.5 to 1 mg/kg with analgesia similar to that of IM [21,22].

Additionally, it was discovered that adult and pediatric ED patients with acute painful disorders responded well to nebulized ketamine at doses between 0.75 and 1.5 mg/kg [23]. A recent randomized clinical trial with 120 patients showed that nebulized ketamine administered at three distinct doses, 0.75, 1, and 1.5 mg/kg, had comparable analgesic effects [24]. Elsaeidy et al. reported in their systematic review that the ketamine and dexmedetomidine combination has lower pain scores, hypoxia episodes, airway blockage, and emerging agitation than the ketamine and propofol combination. They also reported that the ketamine and propofol combination has significantly higher levels of clinician satisfaction; this could be explained by the drug's shortened recovery period and decreased rate of nausea and vomiting [25]. There is another systematic review that assesses the efficacy of ketamine in comparison with benzodiazepine/opioids in emergency medicine. It reports that ketamine and midazolam/fentanyl demonstrated comparable effects on the University of Michigan sedation scale's measure of sedation depth and pain scores during procedures. The Modified Ramsay Sedation Score revealed that the ketamine group had much deeper sedation. The ketamine group experienced a significantly greater incidence of vomiting and nausea, which were the only significant side effects [26]. A randomized controlled trial evaluating ketamine’s effect in conjunction with opioids for acute pain in the ED reported that ketamine was more effective in reducing pain throughout 120 minutes. This led to a decrease in the overall amount of opioids used and fewer repeat doses of analgesia. Although the ketamine group reported more side effects (51% vs. 19%), the adverse effect profile can be tolerated [27].

According to the results of the previous systematic reviews and randomized controlled trials, ketamine is a powerful analgesic used for the control of acute and chronic pain. It is used in SDK doses to reduce the psycho-perceptual adverse effects after surgery. It can be used intravenously, intranasally, and intramuscularly. It has comparable analgesic effects if it is used at three distinct doses 0.75, 1, and 1.5 mg/kg. When it is combined with dexmedetomidine, it has fewer side effects than the ketamine and propofol combination. The ketamine and propofol combination is associated with a short recovery time and a low incidence of nausea and vomiting. When it is used in conjunction with opioids, it has a more powerful analgesic effect and reduces the overall amount of opioids used.

Nitrous oxide is used as a sedative, analgesic, and anxiolytic. It is a colorless, tasteless gas that is inhaled in conjunction with oxygen. It acts through the antagonism of the central nervous system's NMDA receptor and opioid receptor agonism, causing the release of endogenous opioids [28]. Nitrous oxide is a great drug for pain management in the ED since it can be readily titrated. Nitrous oxide is fast to give its effect and can be delivered via facemask or nasal hood. The most popular concentration is 50% to 70% nitrous oxide (30%-50% oxygen) delivered through a continuous flow device or on-demand inhalation mechanism [29,30]. A powerful, secure, and efficient inhalation anesthetic, nitrous oxide offers rapid and adjustable pain relief for a range of extremely painful conditions or procedures carried out in adults and pediatric EDs [29,30]. Complete cardiopulmonary monitoring is necessary when administering nitrous oxide at concentrations of more than 70% or in conjunction with opioids or benzodiazepines. There is a randomized controlled trial evaluating the effect of nitrous oxide in a high dose in combination with IN fentanyl as an analgosedative combination in children. The results indicate no group difference between N2O 70% combined with INF and N2O 70% without INF concerning Face, Leg, Activity, Cry, and Consolability (FLACC) scale score, Modified Behavioural Pain Scale (MBPS) score, and self-reported pain [31]. So, IN fentanyl can be dispensed with the use of a high dose of nitrous oxide as an analgosedative drug.

A derivative of butyrophenone, haloperidol acts by blocking the dopamine receptor (D2-R antagonist). Furthermore, haloperidol reduces the hyperalgesia brought on by long-term opioid usage via binding to the histamine, alpha-2 adrenergic, 5HT-2, and NMDA receptors [32]. A derivative of butyrophenone, droperidol has strong antagonistic effects on dopamine D2 receptors in addition to acting as an A2 adrenoceptor agonist and an antagonist of 5HT-3, muscarinic, and nicotinic receptors [33]. In the ED, haloperidol and droperidol have been utilized as adjuvants for the following conditions: headache [34], gastroparesis and cyclic vomiting syndrome [35], chronic pain not responding to opioids [36], and stomach discomfort linked to cannabis hyperemesis syndrome [37]. Haloperidol: 2.5-5 mg IV, 5-10 mg IM; droperidol: 1.25-2.5 mg IV, 2.5-5 mg IM are examples of traditional dose schedules and administration methods.

Lidocaine decreases hyperalgesia and cerebral sensitization by non-competitively blocking NMDA receptors and voltage-gated sodium channels [38]. Lidocaine produces mild side effects (perioral numbness, dizziness, periorbital, and tinnitus) that are brief and quickly reversible when given IV at a dose of 1 to 1.5 mg/kg throughout 10 to 15 minutes [38,39]. Even though the initial studies on renal colic showed encouraging results [39], later research showed that IV lidocaine was not as effective as IV ketorolac alone or IV ketorolac/lidocaine combination in terms of analgesia [40]. Comparably, when ED patients presented with acute low back pain [41] and stomach pain [42], IV lidocaine did not show any significant pain alleviation.

Table 1 summarizes the anesthetic drugs used to alleviate the pain in the ED.

**Table 1:** Summary of anesthetic drugs used for pain relief during an emergency ppm: parts per million, IM: intramuscular, IV: intravenous, P.O: per oral, IN: intranasal, NSAIDs: non-steroidal anti-inflammatory drugs, ADHD: attention deficit hyperactivity disorder. Table data adapted from references [43-63].

Drug	Dose and Route of Administration	Onset of Action	Duration of Action	Indications	Contraindications	Side Effects
Ketamine [43-45]	IV (low dose for analgesia: 0.1 - 0.3 mg/kg).	within 30 seconds after injection.	5-10 minutes.	It is indicated as the single anesthetic drug used in surgical and diagnostic procedures that aren't needed for skeletal muscle relaxation. It is used for induction of anesthesia before administering further general anesthetics. It is used as a complement to low-potency drugs like nitrous oxide.	Ketamine hydrochloride is contraindicated in people who have demonstrated drug hypersensitivity or in whom a considerable increase in blood pressure would pose a serious risk.	Hypertension; Tachycardia; Respiratory depression; Apnea; Diplopia; Nystagmus; High intraocular pressure; Inflammatory urinary tract symptoms.
	IM (0.45 mg/kg)	within 3 to 4 minutes following injection.	12 to 25 minutes.			
Fentanyl [46-50]	IV (50 - 100 mcg/kg/dose).	Almost Immediate	30-60 minutes	It is used for its short-duration analgesic action as needed in the recovery room during the first few hours after surgery. It is also used as a premedication. Anesthesiologists can use it for the induction and maintenance of anesthesia.	Patients who have experienced anaphylaxis or other fentanyl hypersensitivity are contraindicated to receiving fentanyl citrate injections.	Misuse, abuse, and addiction; Life-threatening respiratory depression; Serotonin syndrome; Cardiovascular depression; Gastrointestinal adverse reactions; Seizures.
	IM (50 - 100 mcg/kg/dose).	7-8 minutes	1-2 hours			
	IN (1 - 2 mcg/kg/dose).	5-10 minutes	1 hour			
	Nebulized (4 mcg/kg).	Almost Immediate	30-60 minutes			
	Transmucosal lozenge( is available at various doses: 12, 25, 50, 75, and 100 mcg/h).	5-15 minutes	Related to blood level			
Propofol [51-53]	IV (100-150 mcg/kg/min).	Within one minute.	5-10 minutes	Propofol is the most often used induction drug for general anesthesia. Propofol can be administered intermittently or continuously to maintain general anesthesia, providing precise control over the depth of anesthesia during the surgical operation.	It is contraindicated in cases of confirmed hypersensitivity, and allergy to eggs, soybeans, or soy.	Hypotension; Apnea lasts 30-60 seconds; Respiratory acidosis during weaning; Hypertriglyceridemia; Itching; Arrhythmia; Cardiac output decreased (concurrent opioid use increases incidence).
Lidocaine [54-55]	Intradermal injection (max recommended dose without epinephrine: 4.5 mg/kg, not to exceed 300 mg per dose).	1 to 3 minutes.	10 minutes	It is a local anesthetic agent that is indicated for the relief of pain.	It is contraindicated in individuals with a history of amide-type local anesthetic sensitivity.	Agitation; Flushing; Itching; Blisters; Bruising; Burning feeling; Skin discoloration; Loss of pigmentation; Swelling; Exfoliation.
	Transdermal application (patches are available at doses of 700mg and 36mg).	3 to 5 minutes	11 hours (following application of 3 patches)			
Morphine [47,56,57]	IV (50-100 mcg/kg/dose). IV Infusion: 10-20 mcg/kg/h.	5- 10 minutes	4-5 hours	It is used for the control of pain that cannot be adequately relieved by alternatives and that necessitates the intravenous injection of an opioid analgesic. The control of pain using intrathecal or epidural methods without the loss of sympathetic, sensory, or motor function.	Severe respiratory depression and acute or severe bronchial asthma. Concurrent use of monoamine oxidase inhibitors (MAOIs) or usage of MAOIs during the last 14 days. intestinal obstruction. Morphine hypersensitivity (such as anaphylaxis).	Dizziness; Drowsiness; Constipation; Nausea; Vomiting; Lightheadedness; Headache; Increased sweating; Urinary retention; Dry mouth.
	IM (50-100 mcg/kg/dose).	10-30 minutes	4-5 hours			
	P.O (0.1-0.2 mg/kg/dose).	~30 minutes	3-5 hours			
Nitrous oxide [47,58-60]	Inhalation (20 ppm).	2-5 minutes	N/A	In addition to treating severe pain, nitrous oxide can be utilized for dental anesthesia, procedural sedation, and general anesthesia. Because of its strong analgesic qualities, nitrous oxide can be helpful in analgesic environments like the emergency room or obstetrical ward.	Chronic obstructive pulmonary disease. Any history of having bleomycin. Nasal obstruction Claustrophobia Uncooperative behavior/cognitive impairment/psychiatric disorders.	Respiratory Depression; Diffusion hypoxia; Postoperative Nausea and Vomiting; Hyperhomocysteinemia; Subacute myeloneuropathy.
Ibuprofen [61,62]	P.O (200 mg, 1 to 2 tablets every 4 to 6 hours while symptoms persist).	Within 30 minutes after ingestion.	4-6 hours	They are used as antipyretic, anti-inflammatory, and analgesic agents. These effects make NSAIDs useful for treating muscle pain during trauma. Topical NSAIDs are most useful for treating pain due to soft-tissue injuries. Parenteral NSAIDs can be administered as a non-opioid analgesic to manage pain and can also reduce fever.	NSAID hypersensitivity. Salicylate hypersensitivity. Patients who have undergone coronary artery bypass graft surgery. During the third trimester of pregnancy	Gastrointestinal problems' HTN Kidney damage; Allergic reactions.
	IV (400 to 800 mg, every 6)	Within minutes after the injection	4-6 hours			
Aspirin [61,62]	for 325 mg tablets, 1 to 2 tablets every 4 hours, or 3 tablets every 6 hours.	Within 15- 30 minutes	4-6 hours			
Naproxen sodium [61,62]	for 220 mg tablets, 1 to 2 tablets every 8 to 12 hours.	20 - 30 minutes	8 - 12 hours			
Diclofenac sodium [61-62]	Topical solution (1.5%). The typical dose depends on the condition being treated.	1- 3 hours.	It takes around 6 - 12 hours, depending on the treated condition.			
Clonidine [63]	Transdermal patch (0.1 mg/day, 0.2 mg/day, 0.3 mg/day).	Start to exert analgesic effect within a few days (2-3 days).	Up to 7 days.	Hypertension. Treatment of ADHD in children. Management of tics commonly found with Tourette syndrome. Adjunct therapy for severing cancer-related pain. As an adjunct in neonatal opioid withdrawal syndrome.	Drug hypersensitivity. Pregnancy. There is an absolute drug interaction contraindication but there are some drugs that require caution during their concomitant use.	Abdominal pain; Headache; Hypotension; Fatigue; Nausea; Emotional instability; Constipation; Xerostomia; Diarrhea; Sexual dysfunction; Dizziness; Sedation
	P.O (0.1 mg).	30 - 60 minutes	6 - 12 hours			
	Epidural infusion (30 mcg/hr).	15-30 minutes after the initiation of infusion.	6-12 hours with continuous infusion.			

Anesthesia management for pain control in burn patients

One of the most important steps in many phases of burn care, from initial evaluation and wound care to surgical procedures, is the provision of anesthetics. In cases with burns, the main objectives of anesthesia are pain management, aiding in medical procedures, and, occasionally, managing breathing. Local anesthetics are frequently used to numb certain areas and enable medical professionals to clean and dress wounds without distressing patients. It is also used to control localized pain, and nerve blocks may also be employed. Whenever surgical operations are required, general anesthesia is usually given. During their course of treatment, burn patients may require numerous surgeries, such as skin grafts, debridement, and reconstructive treatments. When performing burn surgery, anesthesiologists take into account the patient's general condition, the severity of the burn, and any possible side effects, including fluid imbalance or impaired airways [64].

Anesthesia and airway management in emergencies

Anesthesia and advanced airway management in critically ill patients who arrive at the ED are acknowledged as unique issues that carry a substantial risk of death and morbidity [65]. Performing emergency airway care outside the operating room (OR) has a higher risk than performing it in the ED [65,66]. Most patients in the UK and other countries have received physician-directed pre-hospital emergency management, which means there is a rise in the number of patients receiving essential airway procedures and anesthesia before hospital admission. The majority of pre-hospital airway management starts by describing the challenges that come with carrying out interventions effectively in a pre-hospital setting. According to pre-hospital emergency anesthesia systems and governance frameworks, the management of the airway before reaching the hospital may be as successful and effective as that of the emergency room (ER) [67-69].

Anesthetic management of the most common pediatric emergency

In the pediatric population, planned surgeries make up the majority of procedures carried out. Nonetheless, a few surgeries constitute actual emergencies and necessitate an immediate visit to the OR. The anesthesia team may find many of these surgeries challenging for a variety of reasons. The anesthesia team needs to act rapidly to create a preoperative plan for the pediatric emergent patient's induction, maintenance, emergence, and postoperative care [70].

According to the National Safety Council, suffocation due to foreign body (FB) ingestion and aspiration is the fourth most common cause of accidental death for children aged one to six and the third most common cause for infants under the age of one. This is also one of the most difficult conditions a pediatric anesthesiologist may encounter. Usually, bronchoscopy requires general anesthesia to assist in the removal of the FB [71]. The aspiration for an FB is typically organic in origin and involves meals like peanuts, seeds, or other small foods. The right main bronchus has FB embolisms more frequently than the left, whereas the trachea and larynx experience them less frequently. This condition needs a close collaboration between anesthesiologists and surgeons. Anesthesiologists can use spontaneous ventilation in some cases and controlled ventilation in others. Controlled ventilation is often preferred to avoid coughing and dislodgment of the FB [72]. Spontaneous ventilation is chosen by individuals who consider that positive-pressure breathing may cause dislodgment of the foreign item further distally into the bronchial tree. So, it makes the removal of the object more difficult or, worse, causes a “ball-valve” obstruction with clinical deterioration. Another significant benefit mentioned by supporters of spontaneous ventilation is that there is no interruption of breathing as the surgeon is trying to remove the FB while holding the ocular portion of the bronchoscope open [73].

The role of anesthesiologists in trauma management

Trauma care is a complex field that requires a coordinated effort from various healthcare professionals, especially when dealing with injuries that can range from minor wounds to complex multi-system injuries. Patients who have multisystem trauma cause medical complexity due to shock. So, the nature of their physiology is very different from that of patients who undergo elective surgery. Statistics documented that overall morbidity and mortality have increased due to the operative procedures of emergencies [74]. Improved patient outcomes depend on surgeons and anesthesiologists working together as perioperative partners, starting from the initial trauma response activation to the immediate postoperative care. In contrast to other surgical specialties, trauma employs a well-established systematic approach to the initial care and stabilization of trauma patients. The first step in handling trauma is a quick, systematic assessment of the patient called the primary and secondary surveys. The most widely used approach for determining the most serious risks to life using the ABCDEs of the primary survey is Advanced Trauma Life Support (ATLS) [75]. The idea behind the "ABCDEs" of trauma resuscitation is that morbidity and mortality can be decreased by treating hypoxemia and hypotension. The ability to perform advanced airway management is essential for the definitive care of patients with polytrauma or serious injuries. The outcome of management depends on the provider’s ability to expect difficulty and have a safe and efficient plan [76]. In the ER, intubation for trauma accounts for 20% to 30% [77].

Patients with polytrauma are given different anesthetic considerations. In the ER, analgesia and sedation are used for diagnostic procedures and pain control. Regional anesthesia in individuals with trauma. Sedation's function for trauma patients. Management of anesthesia. The preparation of the OR is to be ready to manage the traumatized patients. Blood pressure control must be done in trauma patients. Critical care management in polytrauma patients, and finally, the management of persistent pain after trauma were discussed [78]. Analgesia and sedation that are used in the ER for diagnostic procedures and pain control have been previously discussed. Superior analgesia and a decreased requirement for opioid analgesics are achieved with multi-modal analgesics. It is considered an integration between the use of non-opioid analgesics and localized analgesic treatments. Patients with acute trauma are increasingly managed by using regional anesthetics and analgesic treatments. The increasing safety and utilization of localized approaches as an emergency analgesic and as a means of delivering anesthesia during surgeries can be attributed to the use of ultrasonography. Compared to opioids and sedatives, it is more effective in providing immediate relief from acute pain and providing anesthesia to the specific wounded area. Regional techniques include paravertebral, erector spinae, serratus anterior plane, quadratus lumborum, transversus abdominis plane, rectus abdominis plane block, liposomal bupivacaine infiltration, and non-neuraxial truncal blocks (Table 2) [79,80].

**Table 2:** Summary of techniques for regional anesthesia Tabla data are adapted from reference [81]. TAP: Transversus abdominis plane

Block Type	Indication	Patient Position	Technique of Application
Intercostal	It is used in postoperative pain management for upper abdominal and chest procedures.	Prone with a pillow under the abdomen and a hanging arm on the side of the block.	Catheter placement may be more challenging in obese patients due to extra adipose tissue.
Transversus abdominis	It is used to relieve abdominal pain from cesarean delivery, hysterectomy, or other lower abdominal procedures.	Ideally in the supine position for ultrasound identification of anatomical markers	It can be performed unilaterally or bilaterally at the end of the abdominal surgery, providing coverage from T7 to L1.
Quadratus lumborum	It is used for postoperative pain relief after midline abdominal surgeries.	Ideally, it is administered with the patient in the lateral position.	It provides a larger area of coverage than the TAP block, numbing the area from T4 to L2.
Ilioinguinal	It is often used for the repair of inguinal hernias, and it offers intraoperative pain relief.	The patient lying supine.	Frequently used intraoperatively in conjunction with sedation and supplemental local anesthetic injection at the site

Strategies that can enhance anesthetic management in emergencies

Anesthesiologists have to take the initiative to optimize their patients' health preoperatively. This includes ensuring that patients are in the best possible condition before surgery, evaluating and treating any underlying medical issues, if possible, and optimizing drug regimens. Care delivery must be both standardized and personalized. Grouping patients according to their complexity and risk helps to customize approaches to anesthesia. It is essential to consider data-driven, evidence-based best practices. These procedures give patients structure and guarantee that they regain their best possible level of psychological, cognitive, and functional health. Decisions for anesthesia management are guided in part by the continuous monitoring of hemodynamics, oxygenation, ventilation, and other indicators throughout surgery. Technology developments in clinical monitoring have greatly increased patient safety. Another method that facilitates the performance of anesthesiologists is the use of advanced monitoring technology. These advancements include bedside ultrasound, neuromuscular transmission monitoring systems, and electroencephalogram depth of anesthesia monitoring. Perioperative cardiac monitoring is improved by employing echocardiography to measure ventricular filling, cardiac contractility, and valvular function. Patient safety during intubation and extubation is ensured by the use of advanced supraglottic airway devices, video laryngoscopy, and airway algorithms. Surgical checklists are now standard in ORs, much like they are in the aerospace industry. They enhance patient safety and aid in error prevention [82,83].

## Conclusions

The literature review underscores the critical importance of anesthesia in emergency and trauma surgery. It emphasizes the need for anesthesiologists to manage rapid decision-making, hemodynamic stability, and effective pain control.

Recent advancements in regional anesthesia and multimodal analgesia are promising for improving patient outcomes and reducing complications.
